# Improvements in Attention Following Cognitive Training With the Novel “Decoder” Game on an iPad

**DOI:** 10.3389/fnbeh.2019.00002

**Published:** 2019-01-21

**Authors:** George Savulich, Emily Thorp, Thomas Piercy, Katie A. Peterson, John D. Pickard, Barbara J. Sahakian

**Affiliations:** ^1^Department of Psychiatry and Behavioural and Clinical Neuroscience Institute, School of Clinical Medicine, University of Cambridge, Cambridge, United Kingdom; ^2^Department of Clinical Neurosciences, School of Clinical Medicine, University of Cambridge, Cambridge, United Kingdom

**Keywords:** enhancement of attention, problems of attention/concentration, maintaining flow, cognitive training, Decoder game

## Abstract

Work and study increasingly rely on the use of technologies requiring individuals to switch attention rapidly between emails, texts and tasks. This has led to healthy people having problems of attention and concentration and difficulties getting into the “flow,” which impedes goal attainment and task completion. Possibly related to this, there is an increasing diagnosis of attention deficit hyperactivity disorder (ADHD) and prescriptions of drugs such as methylphenidate. In addition to ADHD, attention is impaired in other neuropsychiatric disorders, such as schizophrenia and in traumatic brain injury (TBI). Based on neuropsychological and neuroimaging evidence, we developed “Decoder,” a novel game for targeted cognitive training of visual sustained attention on an iPad. We aimed to investigate the effects of cognitive training in 75 healthy young adults randomly assigned to a Cognitive Training (8 h of playing Decoder over 4 weeks; *n* = 25), Active Control (8 h of playing Bingo over 4 weeks; *n* = 25) or Passive Control (continuation of activities of daily living; *n* = 25) group. Results indicated that cognitive training with Decoder was superior to both control groups in terms of increased target sensitivity (A’) on the Cambridge Neuropsychological Test Automated Battery Rapid Visual Information processing (CANTAB RVP) test, indicating significantly improved sustained visual attention. Individuals playing Decoder also showed significantly better performance on the Trail Making Test (TMT) compared with those playing Bingo. Significant differences in visual analogue scales were also found between the two gaming groups, such that Decoder received higher ratings of enjoyment, task-related motivation and alertness across all hours of game play. These data suggest that cognitive training with Decoder is an effective non-pharmacological method for enhancing attention in healthy young adults, which could be extended to clinical populations in which attentional problems persist.

## Introduction

Neuropsychiatric disorders are disorders of cognition, motivation and their interaction (Sahakian, 2014). The most common disorder of neurodevelopmental origin is attention deficit hyperactivity disorder (ADHD), which affects up to 1 in 20 children in the United States (Faraone et al., 2003). In 2011, 11% of children aged 4–17 had received a diagnosis of ADHD (i.e., 6.4 million children; Visser et al., 2014). In approximately 80% of children with ADHD, symptoms persist into adolescence, with 60% still having ADHD as an adult (Kessler et al., 2005b). The most prominent cognitive deficit in ADHD is in attention or concentration, which was recognized as a “subtype” of the disorder in DSM-IV (American Psychiatric Association, 1994; Epstein and Loren, 2013), but has changed to a “presentation” in DSM-5 (American Psychiatric Association, 2013). Other problems in ADHD include deficits in working memory and increased impulsivity (Chamberlain et al., 2011, 2017). Although the degree to which core attentional impairments contribute to deficits in other cognitive domains is not well understood, all involve right frontal cortical dysfunction (Clark et al., 2007).

Methylphenidate, a common treatment for ADHD, and other “cognitive-enhancing” drugs such as modafinil are now frequently used at University and in the workplace to stay alert, maintain concentration and increase task-related motivation (Maher, 2008; Sahakian et al., 2015; Brühl and Sahakian, 2016; d’Angelo et al., 2017). A recent publication by Maier et al. (2018) reported the increasing “lifestyle” use of these drugs by healthy people for improving cognitive performance when studying or at work. Sahakian et al. (2015) and Brühl and Sahakian (2016) further reviewed the reasons for this increasing lifestyle use, including competition in a global environment; stress and frequent travel leading to poor quality sleep and jet lag; work where even small mistakes can have major consequences; and getting “into the flow” of work. It has been emphasized that the emergence of new technologies requiring rapid responses to emails and texts and the working on multiple projects simultaneously, that young people including students are having more problems with sustaining attention and frequently become distracted (Gazzaley and Rosen, 2016). This may be why the Care Quality Commission (2016) reported an over 50% increase in prescriptions for methylphenidate over a 5-year period. They attributed this rise to two main factors: increasing prescriptions for diagnosed childhood and adult ADHD and to its potential for diversion and misuse. As attention/concentration is an important cognitive domain for healthy people to function at work, home or University successfully, and because it is a core deficit in ADHD and in other neuropsychiatric disorders such as schizophrenia and in people with traumatic brain injury (TBI), the current study focused on its enhancement through cognitive training using a game. Whereas pharmacological cognitive enhancement raises a number of safety and ethical concerns in healthy people, non-pharmacological strategies offers low-risk, non-invasive alternatives (Savulich et al., 2017a).

Systematic reviews and meta-analyses have shown that cognitive training is an effective behavioral intervention in healthy people and in those with psychiatric disorders, although the degree of generalization to domains other than the one being trained is not always clear (e.g., Wykes et al., 2011; Kelly et al., 2014). Cognitive training is designed to stimulate cognitive function over time, leading to neuroplastic changes and improved functioning of the underlying neural network (Keshavan et al., 2014). In healthy people, cognitive training of working memory has been shown to increase dopamine receptor density and D_1_ binding potential in prefrontal and parietal regions of the brain (McNab et al., 2009; Klingberg, 2010). In healthy older adults, training-related cognitive outcomes have shown associations with increased hippocampal activation (Kirchoff et al., 2012) and white matter integrity in the ventral attention network (Strenziok et al., 2014). Studies from our own laboratory have shown simultaneous effects on cognition and motivation following targeted cognitive training with evidence-based memory games (e.g., “Wizard^1^”), including patient groups with schizophrenia (Sahakian et al., 2015) and amnestic mild cognitive impairment (aMCI; Savulich et al., 2017b). In these studies, gaming technology was used to maximize cognitive training by maintaining high levels of enjoyment and task-related motivation, with no participant dropping out. Higher duration of cognitive training with a game was also associated with better memory outcomes in professional rugby players at high-risk of concussion (Sahakian et al., 2018).

In this study, we present data from healthy people who played a novel game called “Decoder” on an iPad in order to cognitively train sustained attention. Original features are the extensive neuropsychological and neuroimaging evidence-base for this game (Coull et al., 1996; Aron et al., 2003; Hampshire et al., 2010; del Campo et al., 2013; Pironti et al., 2014). In addition, Decoder was developed as a game for cognitive training in collaboration with a games developer to ensure that it maintained motivation with continued play and that it was individually titrated for difficulty. Throughout gameplay, gangs and missions are procedurally generated: each player gets their own unique game world. Each player can unlock regions and progress through the game in their own way. Decoder was designed for play on an iPad with a view to transfer to mobile phones for greater accessibility and utilization in any environment (e.g., at work, home, bus or train). As control groups for the Decoder training group, we included a group that played “Bingo” on an iPad for the same length of time (active control group) and a group that was assessed at baseline and retested at the conclusion of the study (passive control group). We hypothesized that only the group who played the Decoder game would improve their performance following cognitive training as assessed by target sensitivity (A’) on the Cambridge Neuropsychological Test Automated Battery Rapid Visual Information processing (CANTAB RVP) test, a reliable and objective measure of sustained attention. To determine whether cognitive training of concentration had any deleterious effects on rapid shifts of attention, a different form of attention, we also examined performance on the Trail Making Test (TMT; Reitan and Wolfson, 1985). In neuroimaging studies, performance on this task has been shown to activate the left dorsolateral prefrontal cortex and areas involved in motor control (Zakzanis et al., 2005). We predicted that playing Decoder would have no effect on TMT performance.

## Materials and Methods

### Participants

Seventy-five participants were recruited from the local Cambridgeshire area. Inclusion criteria were fluency in English; age 18–30; not currently taking any psychiatric medication or receiving a psychological treatment; and not having a current or past psychiatric diagnosis. All participants were screened on these criteria using the Mini-International Neuropsychiatric Inventory (Lecrubier et al., 1997) and the Adult ADHD Self-Report Scale (Kessler et al., 2005a).

### Neuropsychological Assessment

#### The National Adult Reading Test (Nelson, 1982; NART)

The National Adult Reading test (NART) is a 50-item estimate of premorbid intelligence. Participants are instructed to read aloud 50 words of atypical pronunciation. Higher scores (0–50) indicate more correct responses (i.e., a higher estimate of intelligence).

### Cognitive Measures of Attention

#### CANTAB Rapid Visual Information Processing Test^2^; (RVP)

The RVP test is a measure of visual sustained attention. Participants are asked to detect sequences of digits (e.g., 2-4-6, 3-5-7, 4-6-8). A white box appears in the middle of screen, of which digits from two to nine appear in a pseudo-random order, at a rate of 100 digits per minute. Participants are instructed to press a button every time they detect a sequence. The main outcome measure is A’, a signal detection measure of sensitivity to the target, regardless of response tendency. The duration of the test is approximately 5 min.

#### Trail Making Test (Reitan and Wolfson, 1985; TMT)

The TMT is a measure of attention, visual search, scanning, processing speed and task switching. Participants are asked to connect a set of 25 targets as quickly but as accurately as possible in a sequential order. In Part A, the targets are numbers (e.g., 1, 2, 3, etc.) and in Part B, the targets alternate between numbers and letters (e.g., 1, A, 2, B, 3, C, etc.). Errors are corrected by the experimenter during the task. The main outcome measure is the time to completion (seconds).

### Personality Trait Measures

All participants completed a battery of baseline personality trait measures including the Barratt Impulsiveness Scale (BIS; Barratt, 1994), the Impulsive Behavior Scale (UPPS-P; Whiteside and Lynam, 2001), the Sensation Seeking Scale (SSS; Zuckerman et al., 1964), the Apathy Evaluation Scale (AES; Glenn, 2005), the Beck Depression Inventory (BDI; Beck et al., 1996) and the Behavioral Inhibition/Avoidance Scale (BIS/BAS; Carver and White, 1994).

### Decoder

Decoder was developed in collaboration between the research team including a professional games developer and healthy young adults, with later engagement of patients with acquired brain injuries to ensure that it was fun, motivational, easy to understand and in line with a concept suitable for both healthy samples and clinical samples in line with public and patient involvement in order to enhance uptake. Our target cognitive process for enhancement was sustained attention (i.e., concentration). The object of the game is to assume the role of a Signal Intelligence officer tasked with breaking up global criminal gangs. To do this, the player must decode their communication by engaging in a visual sustained attention task using different combinations of number strings among distractors, in which the success of each mission is rewarded by exposing letters of the next criminal location (with higher scores revealing more letters). Each location generates a maximum of three missions, which are titrated for individual performance in real-time. High levels of enjoyment and motivation were prioritized during development of the game. Immediate engagement is achieved through the use of visual feedback and music to give the game a sense of flow, responsiveness and excitement. To keep the game motivating across several plays, a large number of variants, including short- (e.g., unlocking new regions) and long-term (e.g., earning all ranks) goals, are used. Personalization is achieved through selection of a character and backstory.

### Procedure

This study received full ethical approval from the University of Cambridge Psychology Research ethics committee (reference Pre.2015.092). All participants provided written informed consent. This study comprised a three-group, randomized controlled design, including: the Cognitive Training Group (“Decoder”), the Active Control Group (“Bingo”) and the Passive Control Group (No Game). Potential participants were first screened for inclusion/exclusion criteria by telephone. Invited participants wishing to participant were then asked to attend a baseline testing session, in which they provided basic demographic information and completed the cognitive test battery, followed by all personality trait questionnaires. Participants were also asked about their technology use including the frequency of time spent using the Internet each week, frequency of playing computer games for enjoyment and prior regular use of cognitive training games or applications with the aim of improving cognition. Any participants reporting current use of any cognitive training games or applications were asked to stop throughout the duration of the study. Participants were then randomized to one of three groups.

Participants in the Cognitive Training Group were invited to attend eight, 1 h sessions of supervised cognitive training (i.e., play Decoder on an iPad, as described above). Similarly, participants in the Active Training Group were invited to attend eight, 1 h sessions of supervised Bingo on an iPad. Bingo was selected as the “active control” game because it uses the same type of stimuli as Decoder (i.e., number strings), but does not contain any element of cognitive training of sustained attention. In this version, participants were required to use a very low level of visual search, with reward only given when obtaining five numbers in a row. After each hour of gameplay, participants in the Cognitive Training and Active Control Groups were asked to rate their experience in terms of enjoyment, desire to continue, alertness and positive mood using 10-cm visual analogue scales. Participants in the Passive Control Group were not given access to either game and continued their daily lives as usual.

A maximum of 4 weeks after the baseline testing session, all participants then completed an outcome testing session, which was identical to the baseline testing session in terms of outcome measures.

### Statistical Analyses

Demographic and baseline questionnaire measures were analyzed using one-way analysis of variance (ANOVA) for continuous variables and chi-square for categorical variables. Baseline and outcome variables were analyzed with an analysis of covariance (ANCOVA). As predictors, we used the participant’s baseline scores on the same test (a continuous covariate) and Group (a fixed factor with three levels: Decoder, Bingo and No Game). For the CANTAB RVP, we also included prior regular cognitive training games/applications as a predictor (a fixed factor with two levels: Yes/No). This approach allowed us to assess the effects of gaming (Group) whilst controlling for within-group variance in pre-existing cognitive performance as well as the potential effects of other games/applications with purported cognitive benefits delivered using an iPad or mobile phone. Pairwise comparisons were made between groups following significant effects of gaming. Sidak correction was applied for adjustment of multiple comparisons.

## Results

### Demographic Variables

The Decoder (*n* = 25), Bingo (*n* = 25) and No Game (*n* = 25) groups did not differ in basic demographic variables including age, gender, years in education and premorbid intelligence (Table 1). The groups also did not differ in personality traits including impulsiveness, impulsive behavior, sensation seeking, apathy, depression or behavioral avoidance/inhibition (Table 1). By design, frequencies of Internet use (*X*^2^ = 0.25) and computer game play (*X*^2^ = 0.33) were not significantly different between groups.

**Table 1:** Group demographics and personality trait measures at baseline for each group.

Measures	No game group *n* = 25	Bingo group *n* = 25	Decoder group *n* = 25	Statistic, *p* value
Age (years)	23.20 (±4.92)	24.88 (±7.60)	24.00 (±4.77)	*F*_(2,72)_ = 0.51, *p* = 0.61
Gender (male:female)	15:10	7:18	10:15	*X*^2^ = 5.34, *p* = 0.07
Education (years)	16.24 (±2.08)	16.64 (±2.45)	17.56 (±2.33)	*F*_(2,72)_ = 2.18, *p* = 0.12
Intelligence (NART)	110.92 (±8.30)	112.88 (±9.04)	114.44 (±10.41)	*F*_(2,72)_ = 0.90, *p* = 0.41
ADHD symptom checklist	1.72 (±1.62)	2.20 (±1.68)	2.40 (±1.61)	*F*_(2,72)_ = 1.14, *p* = 0.33
Impulsiveness (BIS)	63.16 (±10.16)	63.88 (±10.95)	63.28 (±10.37)	*F*_(2,72)_ = 0.03, *p* = 0.97
Impulsive behavior (UPPS)	123.40 (±30.49)	131.24 (±20.69)	130.32 (±25.04)	*F*_(2,72)_ = 0.69, *p* = 0.50
Sensation seeking (SSS)	22.36 (±6.05)	21.12 (±5.10)	19.64 (±6.99)	*F*_(2,72)_ = 1.25, *p* = 0.29
Apathy (AES)	11.56 (±7.14)	11.20 (±14.92)	14.92 (±6.89)	*F*_(2,72)_ = 2.23, *p* = 0.16
Depression (BDI-II)	5.84 (±4.92)	5.80 (±4.85)	8.16 (±5.56)	*F*_(2,72)_ = 1.50, *p* = 0.23
Behavioral inhibition/Behavioral activation (BIS/BAS)	60.32 (±6.81)	62.12 (±5.28)	60.88 (±4.77)	*F*_(2,72)_ = 0.66, *p* = 0.52

*Notes: NART, National Adult Reading Test; BIS, Barratt Impulsiveness Scale; UPPS, Impulsive Behavior Scale; SSS, Sensation Seeking Scale; AES, Apathy Evaluation Scale; BDI-II, Beck Depression Inventory-II; BIS/BAS, Behavioral Inhibition/Avoidance Scale*.

### Attention

#### CANTAB RVP

**Figure 1:**
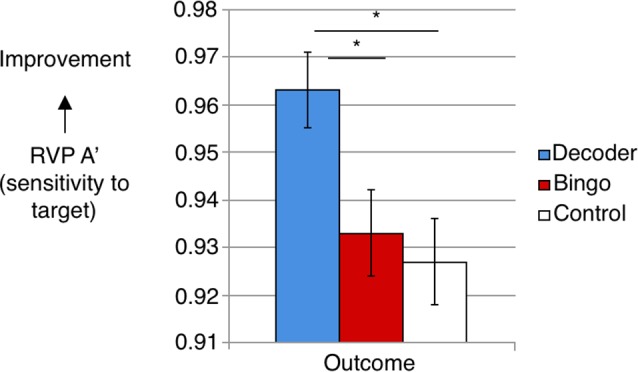
Target sensitivity [Cambridge Neuropsychological Test Automated Battery Rapid Visual Information processing (CANTAB RVP) A’] significantly differed between groups at outcome when controlling for baseline performance, *F*_(2,68)_ = 5.60, *p* = 0.006. The Decoder group showed better detection of target sequences, with adjusted means differing significantly between Decoder and Bingo, *p* = 0.04, and Decoder and Control, *p* = 0.009. *Indicates means significantly differed between groups.

Target sensitivity (A’) significantly differed between groups at outcome, *F*_(2,68)_ = 5.60, *p* = 0.006, *η*^2^ = 0.14 (group means adjusted for baseline performance: Decoder = 0.96, SE = 0.01; Bingo = 0.93, SE = 0.01; No Game = 0.93, SE = 0.01; Figure 1). Pairwise comparisons showed that target sensitivity significantly differed between Decoder and Bingo, *p* = 0.04 and Decoder and No Game, *p* = 0.009, but not between Bingo and No Game, *p* = 0.95. There was an effect of regular cognitive training game/application experience, such that target sensitivity was significantly better in those without prior experience, *p* < 0.001 (group means adjusted for baseline performance: with experience = 0.92, SE = 0.01; without experience = 0.96, SE = 0.96).

#### TMT

**Figure 2:**
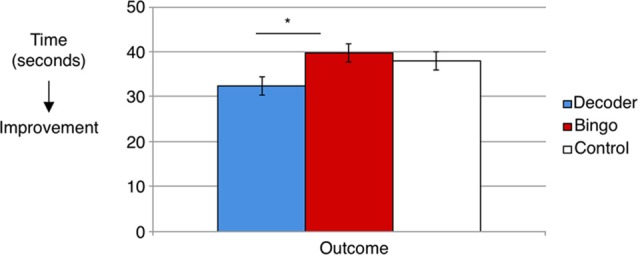
Time (seconds) taken to complete Trail Making Test (TMT) numbers and letters significantly differed between groups at outcome when controlling for baseline performance, *F*_(2,71)_ = 3.72, *p* = 0.03. Adjusted means significantly differed between Decoder and Bingo, *p* = 0.03, such that the Decoder group was faster. *Indicates means significantly differed between groups.

Time (seconds) taken to complete TMT numbers and letters was significantly different between groups at outcome, *F*_(2,71)_ = 3.72, *p* = 0.03, *η*^2^ = 0.06 (group means adjusted for baseline performance: Decoder = 32.28, SE = 2.02; Bingo = 39.73, SE = 2.02; No Game = 37.92, SD = 2.01; Figure 2). Pairwise comparisons showed that time to completion was significantly different between Decoder and Bingo, *p* = 0.03. Time to completion was not significantly different between Decoder and No Game, *p* = 0.15 or Bingo and No Game, *p* = 0.90.

The same pattern of result emerged when including gender as a covariate in the above models; there was no effect of gender for either task (*p* = 0.64 and *p* = 0.65).

### Visual Analogue Scales

**Figure 3:**
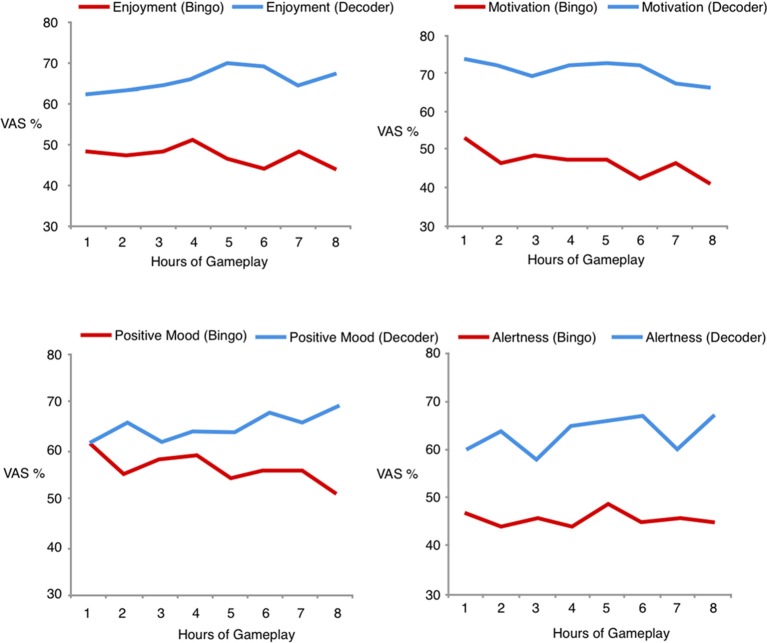
Visual analogue scales for enjoyment, task-related motivation, alertness and mood for Decoder and Bingo across all hours of gameplay.

Across all hours of gameplay, the two game groups significantly differed in their average levels of enjoyment (Decoder = 6.57, SD = 2.08; Bingo = 4.70, SD = 2.18; *t*_(48)_ = 3.11, *p* = 0.003), task-related motivation (Decoder = 7.07, SD = 2.12; Bingo = 4.64, SD = 2.77; *t*_(48)_ = 3.59, *p* = 0.001) and alertness (Decoder = 6.35, SD = 2.12; Bingo = 4.59, SD = 2.18; *t*_(48)_ = 2.90, *p* = 0.006). Positive mood did not significantly differ between the two groups after the 1st hour of game play (Decoder = 6.08, SD = 2.31; Bingo = 6.20, SD = 2.18; *t*_(48)_ = 0.19, *p* = 0.85), but gradually increased for Decoder, and decreased for Bingo, and significantly differed after the 8th h of game play (Decoder = 6.92, SD = 5.12; Bingo = 5.12, SD = 2.20; *t*_(48)_ = 3.05, *p* = 0.004; Figure 3).

## Discussion

The main finding of this study was that attention/concentration was significantly improved by playing the Decoder game on an iPad, which was developed for cognitive training. This enhancement in attentional performance by the Cognitive Training Group was seen both in comparison to the passive control group and also the active control group who played Bingo for the same time period. In addition, subjective measures of enjoyment, motivation, alertness and positive mood remained at high levels in those who played Decoder after every hour of gameplay.

Attention prioritizes sensory processing according to task relevance (Sarter et al., 2001). Focused attention involves the ability to respond to specific stimuli (Commodari, 2017). It plays a key role in many cognitive functions such as problem solving and reasoning (Sohlberg and Mather, 1989). Divided attention refers to the optimal allocation of resources between different sets of input by rapid shifting of attentional focus, in an attempt to process information in parallel from multiple sources (Parasuraman, 1998). Different attentional processes have separate, but also overlapping neural networks (Nebel et al., 2005; Petersen and Posner, 2012; Esterman et al., 2014). It is possible that by improving one form of attention that it is at the expense of impairing another form of attention. Gazzaley and Rosen (2016) have argued that current styles of working and the multifaceted way that we engage with the environment is affecting our ability to sustain attention under conditions of distraction and interruptions which are often technology-related. Importantly, training of sustained attention/concentration did not impair performance on the TMT, which favors shifts in attention. Indeed, if anything, gameplay had a beneficial effect on this form of attention also. Decoder in the present study was aimed at healthy young adults wanting to enhance their attention or in those who easily get distracted. While we used iPads, the Decoder game will be available for all iPhone and iPad users of Peak Brain Training^3^ in January 2019. In addition, it has been noted that healthy women have attentional problems specifically associated with the menstrual phase (Merritt et al., 2007; Pletzer et al., 2014, 2017), which might be improved by attention/concentration training.

While healthy young adults were used as participants in the current study, the aim of cognitive training with Decoder for future studies is to assess whether it can improve attentional performance, a key cognitive domain that is impaired in ADHD, schizophrenia and brain injury (Pironti et al., 2014; Lustig and Sarter, 2016; Savulich et al., 2018). The CANTAB RVP test, the main outcome of the present study, has been shown to utilize a fronto-parietal neural network (Coull et al., 1996). Using this test, attention has been shown to be a cognitive endophenotype in ADHD, as performance was impaired in both adult patients with ADHD and also their unaffected first degree relatives, compared with healthy volunteers (Pironti et al., 2014). Furthermore, neuroanatomical abnormalities in gray matter volume in the right inferior frontal gyrus and white matter volume in the caudal portion of the right inferior frontal gyrus and fronto-occipital fasciculus were shared between patients with ADHD and their unaffected relatives (Pironti et al., 2014). Performance on the CANTAB RVP test has also shown to be a useful cognitive endophenotype for ADHD genetic studies over and above other tasks of motor speed and visual search (Gau and Huang, 2014). In neuroimaging and lesion studies in healthy people and in patients with ADHD, the right inferior frontal gyrus has been identified as a key area in the neural circuitry underlying response inhibition (Aron et al., 2003; Hampshire et al., 2010; Fan et al., 2018). Future research could determine the underlying neural network changes following attention/concentration and flow training with Decoder. For children with ADHD, there are a few games aimed at self-regulation and social cognition (Prins et al., 2013; Bul et al., 2015).

In addition to ADHD, attentional function is commonly impaired following concussion and TBI (Stierwalt and Murray, 2002). Patients with TBI have been shown to have impairments in sustained attention as measured by the CANTAB RVP (Salmond et al., 2005; Manktelow et al., 2017). The gamification of cognitive training would also have advantages for patients with problems of apathy or negative symptoms, as it can maintain motivation during the training period (see e.g., Sahakian et al., 2015; Savulich et al., 2017b).

In conclusion, we have demonstrated significant improvements in healthy young adults in attention following cognitive training with a game on an iPad developed specifically for this purpose, using evidence-based neuropsychological and neuroimaging studies. It may be that this will be a suitable game for those healthy individuals who find sustaining attention difficult in the workplace and will assist them in maintaining the “flow” despite distraction and interference in the environment.

## Author Contributions

ET, GS and KP recruited and tested the participants. TP and BS designed the Decoder game and TP programmed it. GS and BS analyzed and interpreted the data. BS and JP supervised the study. All authors wrote the manuscript.

## Conflict of Interest Statement

BS consults for Cambridge Cognition and Peak. We have technology-transferred the App to Cambridge Enterprise who intends to technology-transfer the App to the games company Peak so that it can become widely available for use on mobile devices. This has not occurred yet. The remaining authors declare that the research was conducted in the absence of any commercial or financial relationships that could be construed as a potential conflict of interest.

## Footnotes

^1^www.peak.net/advanced-training

^2^www.cambridgecognition.com

^3^www.peak.net
